# Atlanta Academy of Medicine

**Published:** 1874-07

**Authors:** 


### Atlanta Academy of Medicine.

A. W. CALHOUN, M.D., Reporter.

Atlanta, May 4, 1874.

Dr. W. S. Armstrong, President, presiding.

Dr. DeWitt read his essay on Malarial Hæmaturia, as occurring in Southwestern Georgia.

Dr. Taliaferro had heard this subject freely discussed at the recent meeting of the State Association in Thomasville and Bainbridge, where the disease had prevailed to an alarming extent, and had observed a wide difference of opinion as to the character of the urine, whether it contained blood or not.

Dr. DeWitt stated that in one of his cases the urine was passed upon the floor, and upon drying evident clots of blood were found. Had, however, made no microscopic examination of the fluid.

Dr. Taliaferro remarked that if this disease is malarial, it is singular that it does not occur in all malarious regions. As yet it has only been met with in certain districts. He was called a month ago to a child, who had, as he supposed, meningitis. The child recovered and was discharged. A few days since was again called, and found the child walking "to one side" and complaining of pain just below left axilla. The side of the thorax was depressed and the spinal column curved to the opposite side. The sternum was bulged forwards. Asks the opinion of the Academy as to the cause of this deformity.

Dr. W. F. Westmoreland asked if the curvature of the spine could be straightened. In actual disease of the bones of the vertebral column the straightening is difficult if not impossible. When due to paralysis it can often be put to rights.

Dr. Taliaferro replied that the vertebral column could not be straightened in this case.

Dr. Battey reported that his case of vaginal ovariotomy was doing well. She was up and going about.

Dr. Baird had a negro man to apply to him in great distress, growing out of the fact that he could not have sexual intercourse with his wife. The desires and the power of erection were perfect, but immediately upon contact the penis became flaccid. He had been married three years. Had gonorrhœa five years ago. This trouble had existed for three months.

Dr. J. T. Johnson asked if stricture of the urethra was present. Several years ago he had a case with somewhat similar symptoms, and upon using bougies and overcoming a slight stricture the unpleasantness disappeared.

Dr. W. F. Westmoreland had a negro to consult him for the inability of bringing about an erection of the penis. A stricture was discovered, and upon breaking it down erection was readily induced. Thinks perhaps the case of Dr. Baird is at least complicated with stricture.

A negro called upon Dr. Taliaferro, and stated that he was unable to have connection on account of having neither desire nor erection. No history of a previous gonorrhœa, but patient had once fallen and injured himself to the extent of producing bloody urine. Since that time no erection. Upon passing a bougie a tender, irritable point was found, but no stricture. After a half dozen introductions of the bougie the man was fully restored to his usual sexual desires, and his penis to perfect erection.

Dr. W. F. Westmoreland was called to a neighboring town to consult upon the case of a lady suffering greatly, mentally, from some sexual disability. She was thirty years old; had been ten years married; had never menstruated, nor had any of the well-known symptoms following a failure to menstruate. Upon examination no vagina existed—merely a cul-de-sac. Through the rectum the rudiments of a uterus could be detected, with ligaments attached to either side. With a catheter in the vagina, or cul-de-sac, and the finger in the rectum, simply the ordinary thickness of the walls of the two canals was discovered. As a consequence of several former operations, the rectum had been considerably cut. The mammary glands are well developed; in short, she is, to all outward appearance, a perfect woman, having all the desires and enjoyments incident to married life that other and healthy women enjoy. Yet never a symptom of menstruation has made its appearance; no flushes, no headaches, etc. She has recently discovered her true condition, and is now, mentally, deeply disturbed. He has seen another somewhat similar case in this city, but in this there was not even the trace of a uterus. She had, however, monthly all the symptoms dependent upon interrupted menstruation.

Dr. Armstrong had three times in his professional life met with analagous cases, one of which was in the hands of Dr. Baird. In each case there was a short cul-de-sac, as also rudiments of a uterus. The vagina simply ended in the cul-de-sac. At the time of seeing them each was perfectly well, having sexual desires. They had their monthly troubles from disturbed menstruation. There was no connection between the rudimentary uterus and the vagina, or cul-de-sac, but they were distinctly separate. Since the time of this examination two of the cases are in bad health, in consequence of menstrual disturbances.

Dr. Battey was consulted years ago by a married lady who was barren, but desired issue. Her health was apparently perfect, but there was no appearance of the menstrual molimen. Had been married six or seven years. Examination discovered nothing wrong. Mammary glands normal; everything right; complained of nothing but barrenness. There was nothing peculiar but absence of the menstrual molimen. The whole question of the physiology of menstruation is undergoing thorough investigation, and it is not at all improbable that we may have to change some of our existing ideas upon the subject. The removal of the ovaries does not impair the aphrodisiac propensities. This is certain, that the removal of both ovaries does not always interrupt the menstrual habit; that is, the regular discharge of blood from the uterus.

Dr. Taliaferro believed the case of Dr. W. F. Westmoreland had ovaries, as she was thoroughly developed, but thought the ovaries were undeveloped.

From his reading upon the subject of ovulation, Dr. W. F. Westmoreland was impressed with the belief that much is to be learned in reference to it, from comparative physiology. He is sure it will be found that our ideas upon the work of the ovaries, menstruation, etc., will soon be radically altered.

---

JAMES B. BAIRD, M.D., REPORTER.

---

ATLANTA, May 11, 1874.

The President, Dr. W. S. Armstrong, presiding.

Dr. Battey read an essay on Intestinal Obstructions—a safe and ready method of treatment. The treatment advocated was distensile enemata of tepid water. A number of cases were cited to show the range and efficacy of the mode of treatment, in two of which the injection was vomited by the stomach; and a recent experiment of the writer was detailed, which proved that water could be injected into the rectum of a cadaver until it flowed from the mouth, having traversed the entire alimentary canal. The Doctor illustrated his views, in the presence of the Academy, by forcing air into a section of the small intestine. Knots made in the gut were readily untied, and the intestine, grasped firmly between the fingers, was extricated with facility, simply by breathing into the gut, demonstrating the manner in which it is freed from constricting bands or hernial incarceration. The caput coli with the ilio cæcal valve was exhibited, and it was clearly shown that pressure from within outwards, upon the walls of the cæcum separated the sides of the valve, and allowed the fluid to pass upward through the orifice.

Dr. W. F. Westmoreland remarked that he recognized the importance of the plan of treatment set forth in the essay, but felt confident that obstructions may be met with that so mild a remedy will not overcome. It may be useful in invagination and knot, but thought in strangulated hernia the inflation on the opposite side of the stricture and early adhesions will render it inefficient. In reference to the experiments performed, he spoke of the difference between the normal and abnormal conditions of the intestines, and expressed his fears that the claims of the method as a therapeutic means would be injured by claiming too much for it.

Dr. Battey replied that he thought Dr. W. under-estimated the force that could be exerted in this way. There is scarcely a limit to the power of hydraulic pressure. The pressure of an incompressible liquid forced into the canal is immense, and the question which presents itself is, will the obstruction be removed or rupture occur? When he considers the force that may be thus exerted, in addition to taxis, etc., in strangulated hernia, he is encouraged to hope that in a great degree the necessity for the knife may be obviated.

Dr. W. F. Westmoreland reported the following case: A lady, æt 30. Diagnosis, ovarian (?) tumor about four by eight inches in diameter, without much attachment. This opinion was concurred in by Dr. McDowell, of Barnesville, and Dr. Banks, of Griffin. The tumor is movable in every direction, except upward, and the attachment seemed to be in the left iliac region. The uterus was separately movable; hence the possibility of the tumor being a fibroid of the uterus was excluded. The dragging sensation in the pelvis, which she experienced in the erect posture, had forced her to keep her bed for a number of months, though she is apparently in good health otherwise. Operation: On Friday, 8th inst., she was etherized, and in the presence of a number of gentlemen an incision, four inches in length, was made through the abdominal walls. The tumor, to the feel, was solid, firm and dense. The incision in the peritoneum was enlarged and three fingers introduced into its cavity for the purpose of exploring the tumor. To his surprise and mortification, extensive attachments were encountered. An artery, which from its pulsation he judged to be as large as the radial, passed into the tumor posteriorly. The tumor rolled easily from side to side, over the spinal column, but could not be moved directly upward or brought forward through the incision. The wound was closed, and the tumor allowed to remain. A telegram received to-day, seventy-two hours after the operation, informed him that the patient was doing well—no unpleasant symptoms having appeared. He entertains doubts as to the nature of the tumor.

Dr. Battey inquired if there was any appearance of an ovarian or malignant cachexia?

Dr. Westmoreland answered in the negative, and said that she had the appearance of perfect health, though her menstrual functions had been much interfered with.

Dr. DuPre suggested that the pain described by Dr. W. indicated the probable location and extent of the attachments, as ovarian tumors much larger than the tumor under discussion, do not cause such disturbance.

Dr. Battey added that the symptoms might not only indicate the character of the attachments, but also that an important organ was involved.

Dr. Westmoreland said that in this particular case the pain was caused by pressure of the tumor on the uterus, as in the erect position the womb was in a state of complete retroversion.

---

A. W. CALHOUN, M.D., REPORTER.

---

ATLANTA, May 18, 1874.

Dr. W. S. Armstrong, President, presiding.

Dr. Battey read a short history of the united twins demonstrated to the Academy several weeks since. They were not symmetrical, one being a little higher than the other. It is probable that the higher one first escaped by the head, and the second, sinking in upon the first, followed immediately afterwards.

Dr. B. also related a case, with the view of having an expression from members of the Academy as to the diagnosis. A physician discovered in his wife (now thirty-two years old), an enlargement in the left hypochondriac region, and supposed it was an enlarged spleen. This was sixteen years ago. It slowly increased in size, and gave some pain, but it was the source of no anxiety, the wife regarding it as "a simple flat hardness in her side." Six years ago she suffered from a severe attack of illness, and had a good deal of soreness in this region. During this time she felt that something tore loose from the side, and at once the tumor fell downwards and towards the right. During the interval from then until now her general health has failed; has frequent acute pains of variable duration, but is herself not fully satisfied as to the exact seat of the pain. Menses appear regularly, with occasional hemorrhagic tendency, after which the pain diminishes and the tumor grows smaller.

Upon examining the case yesterday the tumor was felt a little to the right of the median line, solid, smooth and regular in its contour, reminding one of the liver. One of its edges is sharp and well defined; its right aspect being rather concave, not dissimilar to half a watermelon, somewhat hollowed out on its face. It reaches from about the level of the false ribs down to the superior strait of the pelvis, and is in nowise connected with the uterus, each being movable without interfering with the other. There is a leucorrhœal discharge, and the uterus is retroverted, with corporeal endometritis, all of which can account for the menorrhagia. The tumor is apparently supported from above and below, (patient in recumbent position), and quite movable upon the anterior parietes of the abdomen. Difficulty was found in examining patient in knee-elbow position, as vertigo ensued, and the examination could not be satisfactorily continued. This movable condition of the solid body did not divest the mind of the idea of adhesions. In consequence of the abdomen being distended by the mass, the ovaries could not be clearly located. The intestines were displaced to the right side, and a hollow, tympanitic sound followed percussion over the region of the spleen and the right side. The question is, "what is it?" The physicians at her home diagnosticated it to be an ovarian tumor, and she is sent here with the view of being relieved by an operation, in case the diagnosis is confirmed and surgical interference is deemed advisable. Attention was again called to the remarkable fact that of Spencer Wells' thirteen cases of ovariotomy, with *extensive and formidable adhesions*, requiring severe operations, all recovered with but one exception. Amongst a number of similar cases quoted from Atlee there was not a single fatal instance.

Dr. Armstrong demonstrated a pathological specimen. Was called a few nights back to see a laborer forty-eight years old, who had been sick ten or twelve days without treatment. He was delirious, pulse about ninety, skin of the usual temperature, tongue furred, pupils slightly dilated and not responsive to the influence of light. Had complained of headache during ten or twelve days. The doctor was at a loss to know the nature of the case exactly, but was induced to settle upon a diagnosis of meningeal trouble. Gave him a purgative of calomel and podophylin and blistered his scalp and neck, and found him better next morning. Oil was given to aid the purgative dose. The patient became conscious in the afternoon. Oil was given a second time, as the medicine had not yet acted, nor had he passed urine. Gave an enema the next day, with some effect. Until this time he was conscious, but now came on pain in the extremeties, with delirium. There was no intolerance of light, as in the beginning. Patient died last night, and post-mortem was made this morning, assisted by Dr. Baird. Slight congestion existed upon various portions of the surface of the brain, and at certain points little specks or deposits of exudation, indicative of meningitis. Has found each year less and less of this adventitious matter in meningitis. Thinks the slight symptoms of the disease in this case due to the small amount of these apparently tubercular deposits.

Dr. W. F. Westmoreland had a case from a neighboring county of supposed ovarian tumor. Was assisted in the examination by Drs. Battey, Logan and Taliaferro. Two years ago she bore a child, the after-birth not coming away for some considerable time afterwards. During the interval between the birth of the child and the delivery of the placenta there was much pelvic inflammation. Six weeks later she detected a tumor in the abdomen, which after three months suddenly grew smaller, and a quantity of pus passed away by the rectum. Upon examination the uterus was pushed downwards; there was ante-flexion and ante-version. The tumor felt quite as large as a natural uterus. With fingers in the rectum and vagina, it was seen that the enlarged cervix uteri was forced backwards against the rectum. After each examination the patient had a free discharge of pus by the bowels. What was the original difficulty? Perhaps the uterus was acutely inflamed and an abscess followed, implicating the adjacent structures, and finally breaking into and discharging through the rectum. There were paroxysms of pain whenever the amount of pus became scanty, being again relieved by a free flow. Could not the abscess be punctured through the walls of the abdomen, and give the pus a direct outlet? The great difficulty in the way is that the exact position of the cyst could not be definitely ascertained. It was decided to send her home to build up her health, in the hope of a spontaneous cure. Mentions several similar cases where the abscess broke into the rectum, and in some of them spontaneous cures followed.

Dr. Battey has had some cases of peri-uterine cellulitis with consequent abscess, and, without exception, each recovered in a comparatively short time. A few have discharged into the rectum, one through the vagina, and two into the pelvis of the kidney, passing off with the urine. Has had no case of long-continued purulent discharge. His opinion in regard to Dr. W.'s case is that most of the tumor consisted of the uterus and peri-uterine cellular tissue, which, becoming inflamed, formed an abscess, burst and discharged into and through the rectum. Thinks this mode of outlet less favorable than by the vagina. In this case nothing more could be done than build up the system and leave the cure to nature.

Dr. Woodhull recollected the details of a case related to him years ago, in which the original cause of the trouble was extra uterine pregnancy. Abscess resulted and broke through the rectum, and the decomposed fœtus was passed off with a quantity of pus. The patient recovered.

Dr. Taliaferro's cases have mostly discharged the pus through the rectum. Thinks Dr. W.'s case was metritis and peritonitis, and with it extensive deposits of lymph, and intestinal adhesion. In his opinion the abscess was within the folds of the broad ligament, and an operation was unjustifiable.

Dr. W. F. Westmoreland mentioned another case where openings took place in the bowel, bladder and externally. When any one of these openings was closed the fæcal matter passed through one or both of the others. Seeds and other foreign substances had passed through the bladder.

In reply to Dr. Battey's inquiry about the case of shallow vagina ending in a cul-de-sac, reported at a previous meeting by Dr. W. F. Westmoreland, the latter answered that nothing further had been developed, except upon second examination both ovaries were discovered and a rudimentary uterus. He was mistaken in his statement about the woman having had no menstrual molimen. She now thinks she has "not been well" since her fifteenth year, but thought all the while there was no connection of this monthly indisposition with any malformation. Patient was told that no operative treatment was justifiable.

Dr. Battey was under the impression that the case reported by himself was one of enormously hypotrophied spleen. Atlee reports two cases, and made abdominal section in one for ovarian tumor. In neither of Atlee's cases did the solid tumor go down into the pelvis, as in his (Dr. B.'s), where it was fastened. The supposition is that six years ago the spleen, on account of its extreme weight, tore loose in part from its natural attachments and fell into the pelvis, and there became attached.

Dr. Woodhull could not see what else this enlargement could be, aside from a dislocated spleen. There are several reports on record of such dislocations of the spleen.

---

Atlanta, May 18, 1874.

Dr. W. S. Armstrong, President, presiding.

Dr. Logan makes mention of a conversation had with a physician living in the malarial district of Alabama in reference to malarial hæmaturia (bloody urine), and his successful treatment of the disease by the use of hydrate of chloral.

By request of Dr. Logan, the doctor gave the following hurried note relating to his management of a certain number of cases by the administration of the above-named medicine:

"Note.—Malarial hæmaturia, case 1. Remedies used in the treatment, calomel, quinine, opiates, tincture of iron and mineral acids. Terminated fatally. Cases 2 and 3 had about the same treatment, with like result. Case 4 had the same for four or five days from the attack. The symptoms became very grave, patient delirious, respiration rapid and labored, very restless, had slept none for a day and night. Twenty grains of chloral was administered at once, and not only had the desired hypnotic effect, but arrested the hæmaturia also, which did not return any more. Since this I have seen several other cases of this disease, all of which recovered, though some of the gravest character, moderate doses of the chloral never failing to moderate and finally to arrest entirely the hæmaturia. The remainder of the treatment consisted almost solely in keeping the patient under the influence of the tincture of iron and quinine."

Dr. Battey read the following letter from a professional correspondent, asking advice upon a doubtful case: "A man aged 35, married, called on me two weeks ago, complaining of two little watery pimples upon the penis, with itching and burning. He asserted that he had not cohabited with any female of doubtful character. I did not attach importance to the case, but ordered a dose of salts and cauterized well the pimples. In a few days he returned, the penis covered with sores, also running sores in the beard, having somewhat the resemblance of barbers' itch; a similar eruption likewise in the scalp and upon the thighs. What is it? Direct me."

Dr. Battey did not think the case venerial in character, but a non-specific cutaneous eruption. He did not approve the use of the nitrate of silver stick in affections of the penis. Had seen it do much harm and but little good.

Dr. Baird made inquiry as to what Dr. Battey thought of caustics generally in affections of the penis.

Dr. Battey used them guardedly, and preferred strong nitric acid.

Dr. J. T. Johnson agreed with Dr. Battey in reference to the use of caustics. In his judgment many an innocent penis had suffered, and even been destroyed, by the too free handling of such potent remedies.

Dr. Baird had had in charge a case of chancre, where, after applying nitric acid, the area of the sore still spread. The same remedy was several times reapplied with the same results. He finally concluded that the nitric acid had not been freely and thoroughly enough applied at any one time; whereupon it was again vigorously used with success, the sore ceasing to spread, and soon healing up entirely.

Dr. Battey thought the nitrate of silver did not *jugulate* the disease as did the nitric acid. The latter is used in the treatment of hemorrhoids upon the same principle. Has a case of endometritis in the city in which fuming nitric acid is passed into the uterine cavity after the manner of Atthill of Dublin. The fuming is such that the speculum is filled with smoke. In chancroid thinks the fuming nitric acid better than the weaker nitric acid of the stores.

Dr. W. F. Westmoreland has a case of stricture in a man æt. 55. Two months ago a perineal abscess was opened, together with that portion of the urethra touched or surrounded by the abscess. After this the stricture improved. Now finds an almost impermeable stricture and perineum filled with fistulous openings. Failed to reach the bladder, even with the filliform bougie used as a director. The case being an extreme one, the perineal section was made without a guide. A large quantity of pus escaped from the region of the prostate gland. A female catheter was left in the wound for twenty-four hours. Patient recovered from the operation, and ten or twelve days afterwards the introduction of bougies through the urethra was begun. He now passes urine through the natural channel, and the wound is about healed. Mentions another case in which the urethra could not be passed, but made the perineal section at night, by candle light, and let out the urine from the distended bladder. Thinks the cut ought to be made, even though the urethra cannot be traversed by a filliform bougie.

Dr. Armstrong showed a fœtus, and gave a short history of the case. Saw the woman two months after menstruation ceased, and thought her pregnant. Two months later she thought there must be a mistake, as there was no swelling of the abdomen. About this time she was hurt by a fall, but nothing serious resulted. Later she was scalded upon the foot, and at the same time "felt something quiver in her side." The scalded foot got well, and no abortion took place. There was still no enlargement, even a month or two after this. Five months from cessation of menstruation, while taking a walk of a half mile, pain in the back came on, and she found slight stains upon her linen. This also passed away in a short while. In a short time he was called to see her again, and found the contents of the uterus expelled into the vagina. Was the woman pregnant for five months, or did pregnancy possibly take place later? Fœtus is small, and may have ceased to live when the first injury (the fall) took place, and yet there is no decomposition of the fœtus. The membranes were entire.

Dr. W. F. Westmoreland had a case of supposed pregnancy. Woman was of full habit. Later he concluded she was not pregnant, as she became smaller, Was again called after the expulsion of a dead fœtus. Alarming hemorrhage followed the escape of the placenta. How long had the fœtus been dead?

Dr. Battey—Is cessation of movement an evidence of death, and is movement in the abdomen an evidence of true quickening? Had a patient nearly six months pregnant. She is extremely debilitated, has chronic gastritis, vomiting, digestion very imperfect and she is much emaciated. For years she has had chronic endo-metritis. These severe symptoms in pregnancy are not compatible with a good condition of the uterus. Has always found this to be the case. Patient had undergone treatment with benefit for uterine trouble. Fœtal movements had ceased when she came here, and she supposed the uterus should be emptied of the dead fœtus. But it was decided that this should be left to nature, and hence waited several weeks. Found her in labor ten or twelve days ago, after seven months pregnancy. No movements since she came to Atlanta. She was delivered of a seven-months child, well nourished and still living, but has now infantile jaundice. Attention was drawn to the cessation of the fœtal movements, which did not prove the death of the fœtus. Movements in the abdomen are not always evidence of pregnancy. A patient had ceased to menstruate, and supposed herself pregnant, and prepared clothing, etc., for the child. Was called to deliver her. She had pains, but found an unimpregnated uterus, and no evidence of tumor. Movements could be felt in the abdomen, but there was no child in the uterus. A fœtus can remain dead within the uterus for some time without decomposition taking place. Has seen several such instances. In Dr. Armstrong's case the vital powers may not have been sufficient to bring the fœtus up to the full size, when it ultimately died and was expelled. Mentioned a case of apparent death of an adult, where only after three days watching could it be positively told that the patient was dead. This may have been the case with the fœtus, only in the latter this state of doubtful death was longer continued.

Dr. DuPre—A lady, while in transit to some other point, stopped in Nashville, and was attacked with alarming hemorrhage, which was checked by clearing out the uterus and using the per sulph. iron. Clearing the clots from the vagina, found the fœtus, but no placenta. Tamponed and left her, and found placenta next morning. What was remarkable was the size of the six-weeks fœtus. But we know that at this early age we sometimes have the hair, nails, etc., tolerably well developed.

Dr. Tailiaferro—In doubtful pregnancy the pulsation of the fœtal heart is positive proof, and can usually be readily obtained.

Dr. Logan called attention to a case of acute glossitis, after long continued and severe use of the tongue, the patient becoming heated and suddenly chilled. Pain seated in the muscles of the right side of the neck, extending into the jaw. Deglutition was painful. At first was at a loss to know what the disease was. Upon giving an opiate and veratrum he became quiet and easy. On the following morning the tongue was intensely swollen, filling the mouth and pharynx, and obstructing deglutition and articulation. Dr. Battey was called in consultation. Leeches were applied to the tongue and much blood drawn. Next morning all the symptoms were better. Gave a purgative and afterwards quinine, and in a few days patient was convalescent. This is the only case he has met with in a practice of thirty years. Leeches to the tongue is not a common practice. The suggestion was gotten from Aitken. The doctor thinks the life of this patient was saved by leeching.

Drs. Battey, Taliaferro and Calhoun mentioned similar cases having occurred in their practice, in all of which deep incisions were made into the tongue, allowing a free flow of blood and bringing about rapid recovery. In the case of Dr. Calhoun an abscess formed in the center of the tongue, which, being opened, soon got well.

Dr. J. T. Johnson made special mention of the fact that all of the reported cases had occurred in males. This was quite strange, since the disease is often the result of too free a use of the tongue, and as the gentler sex is given to that, why not the same result?

Dr. DuPre thought general venesection might be good, in addition to scarification, leeching, etc.

Dr. Armstrong demonstrated a scirrhus mammary gland, removed the day before, assisted by Drs. H. V. M. Miller and Bell. Patient æt. 50, discovered six years ago a tumor of the size of a walnut. In December last the breast was injured by a fall, when the tumor took on a very rapid growth, with considerable pain. General health good. Axillary glands not implicated, and no cancerous cachexia. Entire gland was removed.

Adjourned.

